# Candidate genes and pathogenesis investigation for sepsis-related acute respiratory distress syndrome based on gene expression profile

**DOI:** 10.1186/s40659-016-0085-4

**Published:** 2016-04-18

**Authors:** Min Wang, Jingjun Yan, Xingxing He, Qiang Zhong, Chengye Zhan, Shusheng Li

**Affiliations:** Department of Emergency and Intensive Care Unit, Tongji Hospital, Tongji Medical College, Huazhong University of Science and Technology, 1095 Jiefang Avenue, Wuhan, 430030 China; Institute of Liver Diseases, Tongji Hospital, Tongji Medical College, Huazhong University of Science and Technology, Wuhan, 430030 China

**Keywords:** Acute respiratory distress syndrome, Sepsis, Differentially expressed mRNAs, Functional enrichment analysis, Pathway analysis, Transcription factors

## Abstract

**Background:**

Acute respiratory distress syndrome (ARDS) is a potentially devastating form of acute inflammatory lung injury as well as a major cause of acute respiratory failure. Although researchers have made significant progresses in elucidating the pathophysiology of this complex syndrome over the years, the absence of a universal detail disease mechanism up until now has led to a series of practical problems for a definitive treatment. This study aimed to predict some genes or pathways associated with sepsis-related ARDS based on a public microarray dataset and to further explore the molecular mechanism of ARDS.

**Results:**

A total of 122 up-regulated DEGs and 91 down-regulated differentially expressed genes (DEGs) were obtained. The up- and down-regulated DEGs were mainly involved in functions like mitotic cell cycle and pathway like cell cycle. Protein–protein interaction network of ARDS analysis revealed 20 hub genes including cyclin B1 (*CCNB1*), cyclin B2 (*CCNB2*) and topoisomerase II alpha (*TOP2A*). A total of seven transcription factors including forkhead box protein M1 (FOXM1) and 30 target genes were revealed in the transcription factor-target gene regulation network. Furthermore, co-cited genes including *CCNB2*-*CCNB1* were revealed in literature mining for the relations ARDS related genes.

**Conclusions:**

Pathways like mitotic cell cycle were closed related with the development of ARDS. Genes including *CCNB1, CCNB2* and *TOP2A*, as well as transcription factors like *FOXM1* might be used as the novel gene therapy targets for sepsis related ARDS.

## Background

Acute respiratory distress syndrome (ARDS) is a potentially devastating form of acute inflammatory lung injury as well as a major cause of acute respiratory failure [1]. Its development leads to a high short-term mortality rate and significant long-term consequences among survivors, such as physical and cognitive impairment [2]. This disease has represented an important and costly public health problem. The predisposing factors of ARDS are numerous and assorted, including sepsis, pneumonia, multiple blood transfusions, lung contusion, aspiration of stomach contents, and drug abuse or overdose [3]. Although researchers have made significant progresses in elucidating the pathophysiology of this complex syndrome over the years [4], the absence of a universal detail disease mechanism up until now has led to a series of practical problems for a definitive treatment [5].

Previous study has indicated that ARDS has a close relation with severe sepsis [6], but little is known about the detail differences between sepsis-related and non-sepsis-related ARDS. Clinical research reveals that sepsis-related ARDS has poorer recovery from lung injury, higher overall disease severity and higher mortality than non-sepsis-related ARDS [7], however, the mechanism leading to the development of ARDS is still unclear.

Over the past few years, considerable work has been done to test the contribution of genetic factors that may increase the risk of developing ARDS. A genome-wide association study by Wang et al. [8] have suggested that the BCL2-associated agonist of cell death (*BAD*) gene is a candidate gene associated with the development of ARDS. Besides, variants in more than 30 genes have been associated with ARDS [9]. For instance, common genetic variation in the angiopoietin-2 (*Ang*-*2*) gene is proved to be associated with increased risk of ARDS [10]. Gong et al. [11] suggested that the mannose binding lectin-2 (*MBL*-*2*) deficiency was associated with increased susceptibility to sepsis and ARDS. Additionally, based on the whole genome expression analysis, Kangelaris et al. [12] believe that the exploration of gene expression differences occurring early in the development of sepsis-related ARDS may further reveal the mechanisms of ARDS. Furthermore, based on the gene expression profiling on peripheral blood from ARDS patients, Dolinay et al. [13] found that the inflammasome pathway and its downstream cytokines play critical roles in ARDS development; Wang et al. [14] found that peptidase inhibitor 3 (*PI3*) may be a useful clinical marker for monitoring the early development of ARDS. Thus, the analysis of potential ARDS related genes and pathways based on gene expression profile may be a breakthrough for the further understanding of ARDS pathological mechanism.

In the present study, a bioinformatics analysis was performed based on a previous mRNA expression profile from patients with sepsis or sepsis-related ARDS, which was provided by Kangelaris et al. [12]. Via the investigation of disease related differentially expressed genes (DEGs) and pathways, we tried to explore the mechanism of the ARDS and to provide valid biological information for further investigation of this devastating disease.

## Methods

### Affymetrix microarray data

The mRNA expression profile of GSE66890 provided by Kangelaris et al. [12] was downloaded from a public functional genomics data repository Gene Expression Omnibus in National Center of Biotechnology Information, based on the platform of GPL6244 [HuGene-1_0-st] Affymetrix Human Gene 1.0 ST Array [transcript (gene) version] (Affymetrix Inc., Santa Clara, California, USA). This profile included 29 whole blood mRNA samples of patients with sepsis-related ARDS, and 28 whole blood mRNA samples of patients with sepsis alone.

### Differential expression analysis

The oligo software [15] in R was used to preprocess the gene expression profile data. The CEL source files were performed background correction, quartile data normalization and calculating expression using robust multi-array average (RMA) algorithm [16] in affy (http://www.bioconductor.org/packages/release/bioc/html/affy.html). The DEGs between sepsis-related ARDS group and sepsis alone control group were analyzed using the limma package (available at http://www.bioconductor.org/packages/release/bioc/html/limma.html) in Bioconductor software [17]. The *t* test was used to identify the *P* value. fold change (FC) was calculated. *P* < 0.05 and |log_2_FC| ≥ 0.4 were defined to be statistically significant.

### Functional enrichment analysis

The database for annotation, visualization and integrated discovery (DAVID, http://david.abcc.ncifcrf.gov/) [18] is a gene functional classification tool that provides a comprehensive set of functional annotation tools for investigators to understand biological meaning behind large list of genes. Gene Ontology (GO, http://www.geneontology.org) [19] function enrichment analysis were performed based on DAVID, which includes three categories: molecular function (MF), biological process (BP) and cellular component (CC). Kyoto encyclopedia of genes and genomes (KEGG, http://www.genome.ad.jp/kegg/) [20] is a database of biological systems which collects the genomic, chemical and systemic functional information. Reactome (http://www.reactome.org) [21] is a free pathway database that provides intuitive bioinformatics tool for basic research, genome analysis, modeling, systems biology and education. KEGG and Reactome pathway enrichment analyses were performed using DAVID as well. *P* value <0.05 was considered as threshold value for functional enrichment analyses.

### Protein–protein interaction (PPI) network construction

Protein–protein interaction (PPI) network are central to most biological processes, which can help to uncover the generic organization principles of functional cellular networks [22]. Search tool for the retrieval of interacting genes/proteins (STRING) [23] is a biological database and web resource of known and predicted protein–protein interactions. In this study, proteins associated with DEGs were selected according to STRING database with combined score >0.4, and then PPI network was visualized using cytoscape (http://www.cytoscape.org/) [24]. Hub-proteins are small number of proteins with many interaction partners, which play an important role in PPI network [25].

Furthermore, to describe the importance of nodes in the PPI network, three methods including degree centrality [26], Betweenness centrality [27] and subgraph centrality [28] were introduced in the present study. The CytoNCA plugin [29] in cytoscape software was used for the calculation of three methods mentioned above. Furthermore, the modules in PPI network were explored using ClusterOne [30] in cytoscape software. *P* value <2.0E−7 was considered as threshold value for the analysis of modules.

### Prediction analysis of transcription factors

To the further study the pathomechanism of ARDS, the analysis between transcription factors and their target genes obtained from PPI network was performed. IRegulon plugin [31] in cytoscape is used to detect transcription factors, motifs and their optimal sets of direct targets from a set of genes. In this study, iRegulon was used to analyze the transcription factors and their related target genes. The minimum identity between orthologous genes was 0.05, while the maximum false discovery rate on motif similarity was 0.001. The normalized enrichment score (NES) >5 was considered as threshold value for the selection of potential relationships.

### Literature mining analysis of ARDS related genes

GenCLiP software (version 2.0, http://ci.smu.edu.cn/GenCLiP2.0/confirm_keywords.php) [32] is used to perform literature mining analysis for human genes and networks. In GenCLiP, the module of literature mining gene networks [32] can construct a gene-network for the input genes and generate sub-networks based on the user defined query terms, at the same time calculate the probability of random occurrence of the networks through random simulation. In the present study, the literature mining gene networks module in GenCLiP was used to analyze the co-cited network of the ARDS-related genes in the previous studies, and the input genes came from the key genes in the PPI network.

## Resutls

### Identification of DEGs

**Fig. 1:**
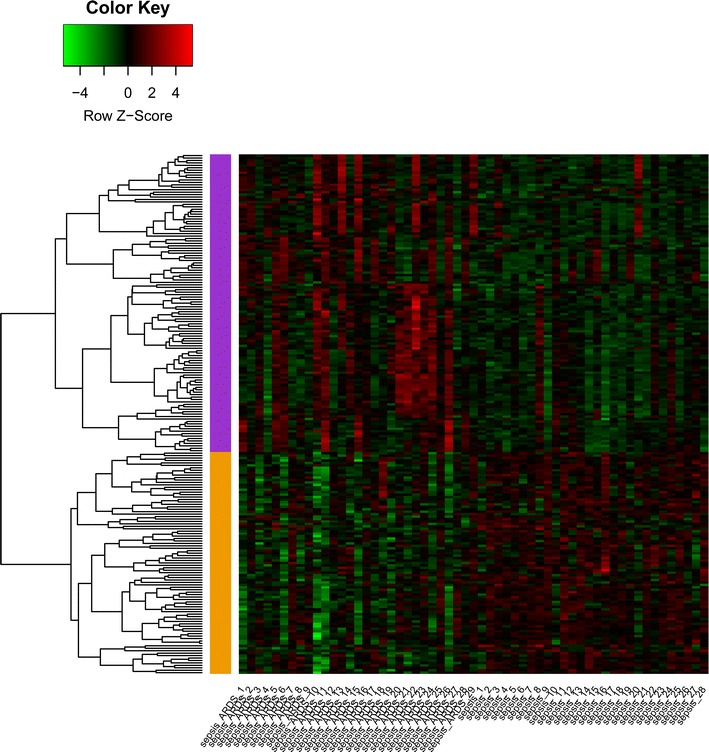
Heat map for the differentially expressed genes (DEGs). *Green* represents the low expression level of DEGs; *red* represents the high expression level of DEGs; *blank* represents the express is not significant

With thresholds of *p* value <0.05 and |log_2_FC| ≥ 0.4, a total of 122 up-regulated and 91 down-regulated DEGs were obtained in sepsis with ARDS group compared with sepsis group. The heat map of differentially expressed mRNAs was showed in Fig. 1.

### Functional enrichment analysis

GO functional enrichment analysis showed that the up-regulated DEGs were mainly involved in mitotic cell cycle (BP, GO: 0000278), cytoplasm (CC, GO: 0005737) and enzyme binding (MF, GO: 0019899). The down-regulated DEGs were mainly involved in positive regulation of response to stimulus (BP, GO: 0048584), spindle (CC, GO: 0005819) and signaling pattern recognition receptor activity (MF, GO: 0008329) (Table 1).

**Table 1:** Results of gene ontology functional enrichment analysis of differentially expressed genes in acute respiratory distress syndrome (ARDS) (Top 12 listed)

		GO ID	Term	Count	P value
BP	Up-regulate	GO:0000278	Mitotic cell cycle	26	1.70E−09
		GO:1903047	Mitotic cell cycle process	24	2.05E−09
	Down-regulate	GO:0048584	Positive regulation of response to stimulus	20	1.31E−05
		GO:0033029	Regulation of neutrophil apoptotic process	2	4.80E−05
CC	Up-regulate	GO:0005737	Cytoplasm	89	5.94E−07
		GO:0005819	Spindle	11	1.56E−06
	Down-regulate	GO:0005819	Spindle	5	0.0055
		GO:0005829	Cytosol	20	0.0092
MF	Up-regulate	GO:0019899	Enzyme binding	26	4.22E−06
		GO:0019900	Kinase binding	12	0.000143101
	Down-regulate	GO:0008329	Signaling pattern recognition receptor activity	2	0.002
		GO:0038187	Pattern recognition receptor activity	2	0.002

P value <0.05 was considered as threshold values of significant difference

*BP* biological process, *MF* molecular function, *CC* cellular component, *GO* gene ontology

The results of pathways enrichment analysis were listed in Table 2. KEGG pathway analysis showed that the up-regulated DEGs were mainly enriched in pathways like Cell cycle, and Hematopoietic cell lineage. The down-regulated DEGs were enriched in three pathways, including phagosome, cytosolic DNA-sensing pathway, and hematopoietic cell lineage. Reactome pathway analysis showed that the up-regulated DEGs were mainly enriched in pathways like mitotic prometaphase, and cell cycle, mitotic; the down-regulated DEGs were enriched in pathways like hydroxycarboxylic acid-binding receptors, innate immune System, and immune system.

**Table 2:** Results of KEGG (Kyoto encyclopedia of genes and genomes) and reactome pathway enrichment analysis of differentially expressed genes in acute respiratory distress syndrome ARDS (top 12 listed)

		ID	Term	Count	P value
KEGG	Up-regulate	04110	Cell cycle	5	0.0035
		04640	Hematopoietic cell lineage	4	0.006
		04114	Oocyte meiosis	4	0.0138
	Down-regulate	04145	Phagosome	4	0.0031
		04623	Cytosolic DNA-sensing pathway	2	0.0216
		04640	Hematopoietic cell lineage	2	0.0496
Reactome	Up-regulate	68877	Mitotic prometaphase	8	4.95E−06
		69278	Cell cycle, mitotic	15	6.51E−06
		2500257	Resolution of sister chromatid cohesion	7	2.81E−05
	Down-regulate	3296197	Hydroxycarboxylic acid-binding receptors	2	6.93E−05
		168249	Innate immune system	10	0.0003
		168256	Immune system	12	0.0011

P value <0.05 was considered as threshold values of significant difference

### PPI network analysis

**Fig. 2:**
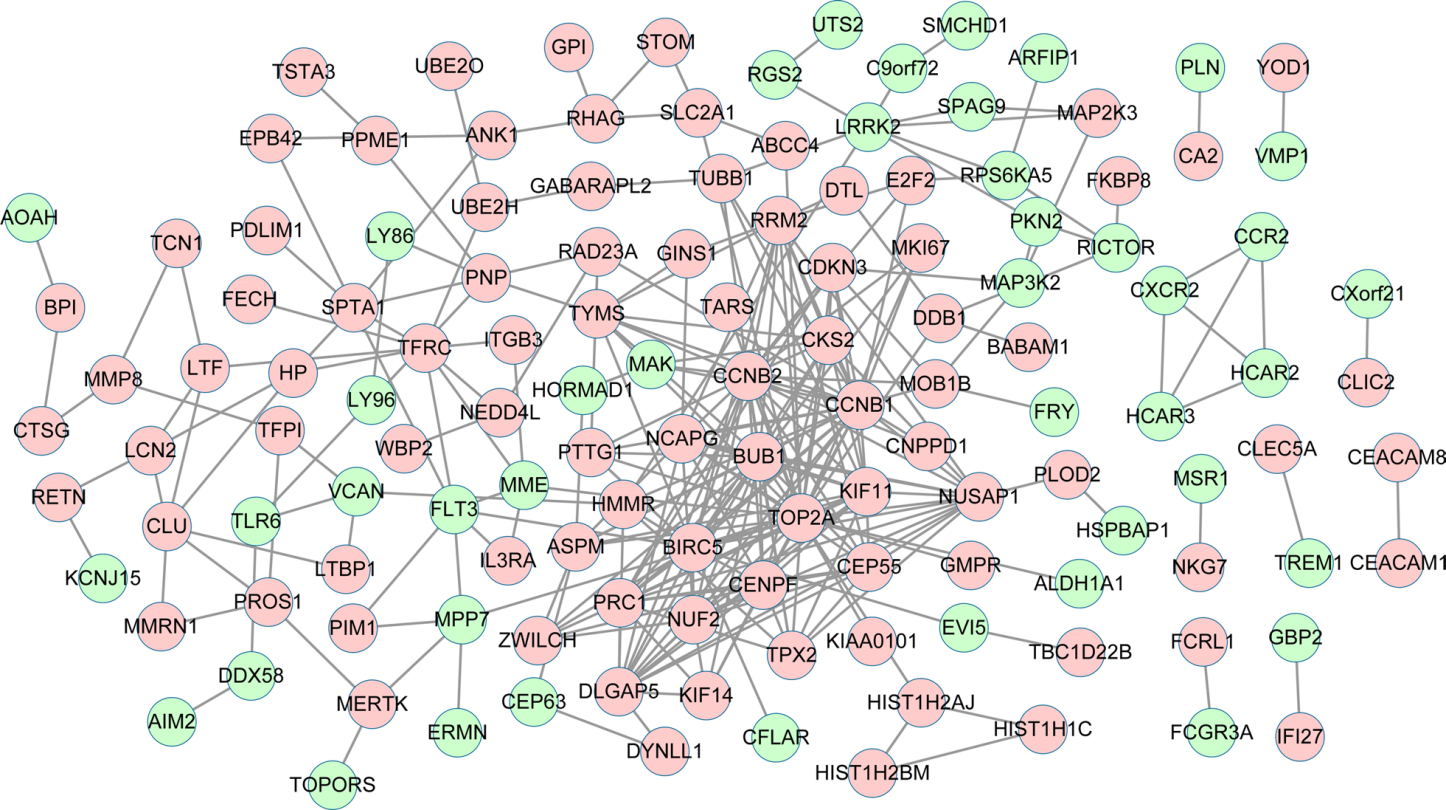
Protein-protein interaction network investigation. *Red* represents the up-regulated gene; *Green* represents the down-regulated gene

**Fig. 3:**
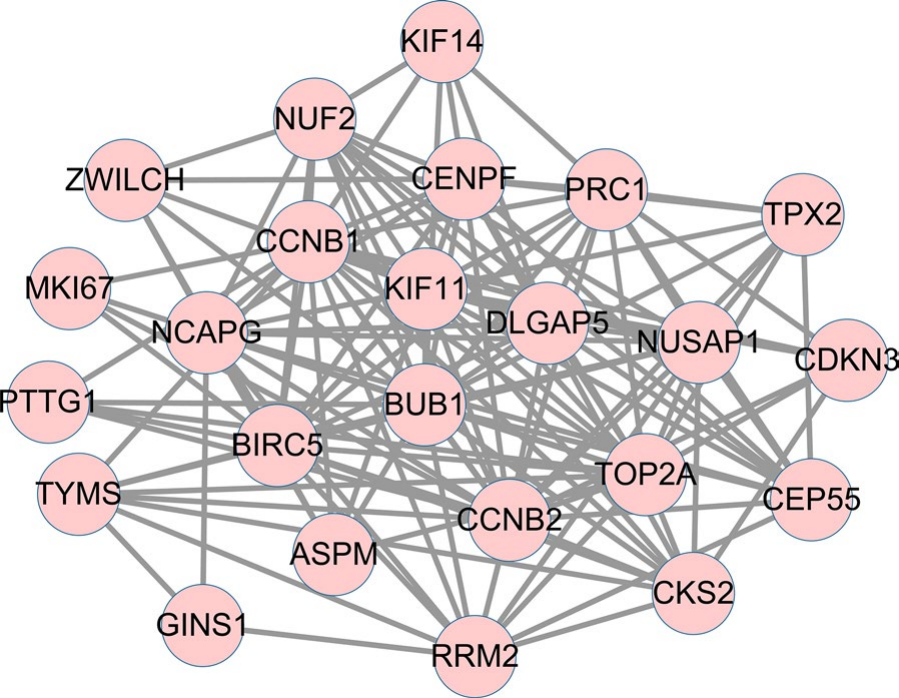
Sub-network module extracted from protein–protein interaction network. *Red* represents the up-regulated gene

With combined score >0.4, a total of 132 nodes with 290 protein interaction pairs were revealed. The PPI network was constructed based on the protein interaction pairs (Fig. 2). Top 20 genes (hub genes) with higher combined score that respectively evaluated by subgraph centrality, betweenness centrality and degree centrality were listed in Table 3. The results showed that cyclin B2 (*CCNB2*) had the highest combined score based on the subgraph centrality evaluation. Meanwhile, the topoisomerase II alpha (*TOP2A*) had the highest combined score in both betweenness and degree centrality evaluations. Furthermore, a sub-network module was obtained from the PPI network (Fig. 3). The result showed that there were 24 genes in the sub-network module, all of which were up-regulated. Interestingly, among the 24 genes, 20 could be found in Table 3.

**Table 3:** Top 20 genes that evaluated by subgraph centrality, betweenness centrality and degree centrality respectively in the protein–protein interaction (PPI) network

Gene name	Subgraph centrality	Gene name	Betweenness centrality	Gene name	Degree centrality
CCNB2	92,020.02	TOP2A	4238.0317	TOP2A	29
CCNB1	91,113.23	HMMR	2151.6792	CCNB1	26
TOP2A	86,794.31	CCNB1	2114.8433	CCNB2	24
BUB1	85,489.84	VCAN	1703.794	BUB1	20
KIF11	72,242.97	BIRC5	1687.0967	BIRC5	20
BIRC5	65,156.9	TFRC	1503.5779	KIF11	18
CENPF	57,783.895	LRRK2	1472.6691	CENPF	15
NUSAP1	57,466.84	TYMS	977.0087	NUSAP1	15
DLGAP5	53,906.652	SPTA1	971.715	DLGAP5	15
NUF2	47,763.83	FLT3	961.2316	NUF2	13
PRC1	46,547.062	MPP7	923.03656	PRC1	13
NCAPG	43,847.594	MME	917.51965	NCAPG	13
CKS2	37,598.816	PNP	807.13245	RRM2	13
CEP55	34,042.55	TFPI	783.21027	CKS2	12
RRM2	32,993.395	RAD23A	767.7287	CEP55	11
TPX2	23,101.832	TUBB1	708.9906	TPX2	9
TYMS	18,167.875	MMP8	698.92865	TYMS	9
CDKN3	14,048.942	CCNB2	688.69775	TFRC	9
KIF14	11,749.522	DDB1	606.85	CDKN3	8
ASPM	11,499.243	LTF	585.95557	ASPM	8

Combined score >0.4 was considered as threshold values of significant difference

### Transcription factor-target gene regulatory network analysis

**Fig. 4:**
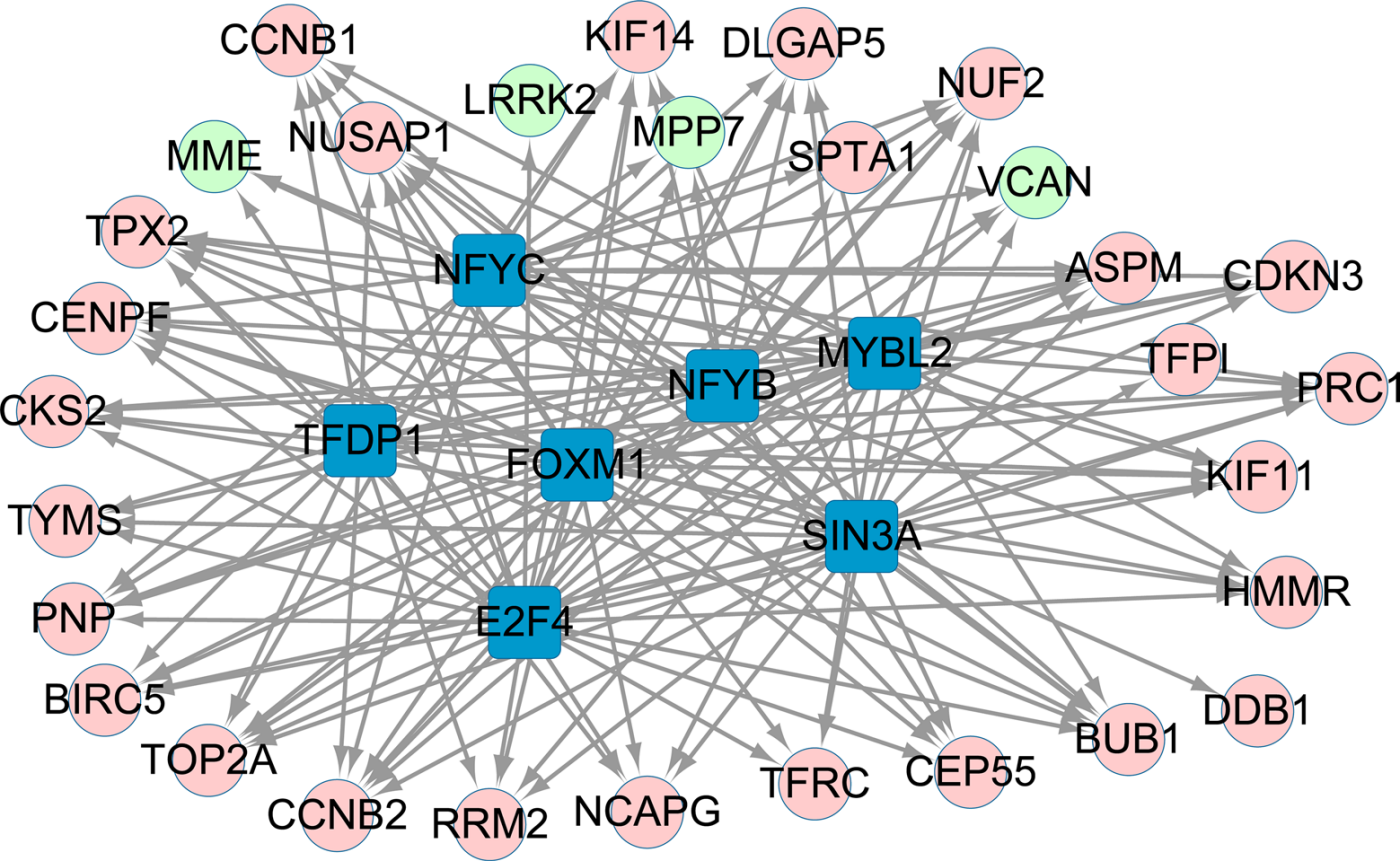
Regulatory network for transcription factors and target genes. *Green circle* represents the down-regulated genes; *Red circle* represents the up-regulated genes; *Blue square* represents the transcription factors

The transcription factors of the top 35 genes with higher scores in Table 3 were predicted. With NES >4, a total of seven transcription factors [such as forkhead box protein M1 (FOXM1)] and 30 target genes [such as hyaluronan-mediated motility receptor (*HMMR*)] were revealed in the present regulatory network (Fig. 4).

### Literature mining analysis

**Fig. 5:**
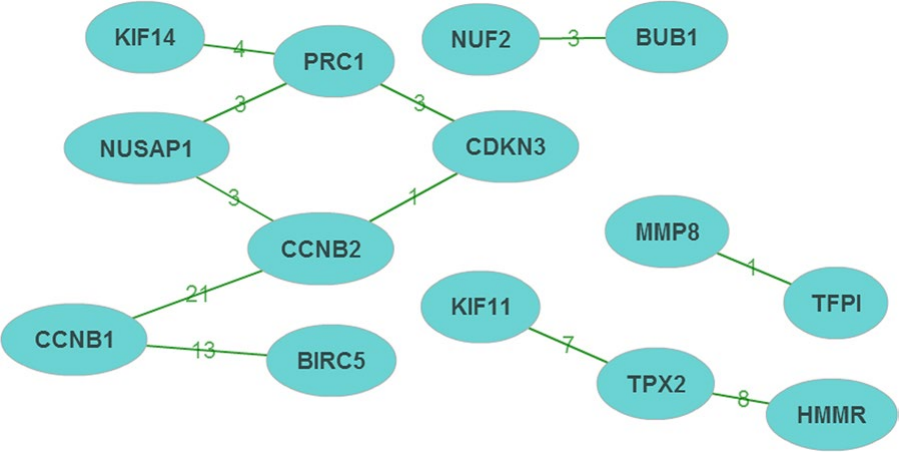
Co-cited network for relations of differentially expressed genes in the previous studies. The number upon on the edge represents the frequency of co-cite for the relation

The result of literature mining analysis revealed 14 genes that were revealed as the key ARDS related genes (Fig. 5). All these genes were differentially expressed in the present study. *CCNB1* and *CCNB2* had the highest co-cited times.

## Discussion

Although intensively and continuously studies have been conducted in ARDS, its mortality is still as high as 30–40 % [33]. In the present study, a bioinformatics analysis between sepsis-related ARDS mRNA samples and human sepsis mRNA samples was performed to explore the mechanism of the ARDS. A total of 122 up-regulated and 91 down-regulated DEGs were obtained. The up- and down-regulated differentially expressed mRNAs were mainly involved in functions like mitotic cell cycle and pathway like cell cycle. PPI network of ARDS analysis revealed 20 hub genes such as *CCNB1*, *CCNB2* and *TOP2A*. In addition, seven transcription factors were revealed in the transcription factor-target gene regulation network. Furthermore, 14 co-cited genes including *CCNB2*–*CCNB1* were revealed in literature mining analysis. These findings may contribute to understanding the development of sepsis-related ARDS.

In the present study, GO functional analysis showed that the up-regulated DEGs, such as *CCNB1*, *CCNB2* and *TOP2A*, were most significantly assembled in BP related to mitotic cell cycle (GO: 0000278, *P* = 1.70E−09) (Table 1). Meanwhile, KEGG analysis also showed that the most significant pathway enriched by up-regulated DEGs was cell cycle (04110, count = 5, *P* = 0.0035) (Table 2). Study has reported that errors in mitosis can either kill a cell through apoptosis or cause mutations which may lead to disease [34]. Therefore, we speculated that some key factors might play important roles in the development of ARDS via taking part in mitosis cell cycle. When and how did these DEGs regulate the process of ARDS? The Reactome pathways analysis showed that mitotic prometaphase (68877, count = 8, P = 4.95E−06) (Table 2) was the most significant pathway enriched by the DEGs. GO analysis showed that both the up- and down-regulated DEGs were assembled in same function like spindle (GO: 0005819). Thus, we speculated that the DEGs might involve in the progression of ARDS via interfering the progress of spindle in premetaphase of mitotic. However, a further investigation is needed to clarify this speculation.

In this study, *CCNB1* and *CCNB2* were two outstanding ARDS-related genes based on the calculation of subgraph centrality in PPI network. Furthermore, the literature mining analyses showed that *CCNB2*–*CCNB1* had the highest co-cited times. As a mitotic cyclin, cyclin B is necessary for the progression of the cells into and out of M phase of the cell cycle [35]. An abnormal cytoplasmic cyclin B1 expression has been found to be associated with a specific T-cell response and cyclin B1-specific immune responses [36]. Importantly, increasing evidences indicate that the immune system plays a key role in lung diseases, including acute lung injury [37]. Activation of the innate immune response by binding of cell injury-associated endogenous molecules to pattern recognition receptors such as the Toll-like receptors on the lung epithelium and alveolar macrophages is now recognized as a potent driving force for ARDS [1]. Taken together, although there is no direct evidence that *CCNB1* and *CCNB2* are involved in ARDS, we speculated that *CCNB1* and *CCNB1* might have close relations in the development of ARDS.

Furthermore, *TOP2A* was also a hub gene had the highest score in betweenness centrality and degree centrality. *TOP2A* encodes a DNA topoisomerase that controls and alters the topologic states of DNA during transcription. Actually, the enzyme of *TOP2A* gene is an essential nuclear enzyme involved in processes such as chromosome condensation and chromatid separation during DNA transcription and replication [38]. Recent study reported that its encoding protein TOP2α is responsible for causing genomic DNA damage [39]. Interestingly, DNA damage is implicated in diverse pulmonary disorders, including acute lung injury [40]. Therefore, we speculated that the up-regulation of *TOP2A* in our study might have a potential relation in the development of ARDS.

The transcription factor-target gene regulation network analysis in this study revealed seven transcription factors including *FOXM1*. *FOXM1* regulates the expression of a large array of G2/M-specific genes including *CCNB2*, and plays an important role in maintenance of chromosomal segregation and genomic stability [41]. In this study, *HMMR* was a target gene of *FOXM1.HMMR* (also identified as CD168) was originally discovered as a soluble protein that altered migratory cell behavior and bound to hyaluronan [42]. HMMR is less well studied than the main hyaluronan receptor of CD44 that has been examined in ARDS secondary to bleomycin injury. Priit et al. [43] have suggested that CD44 plays a role in resolving lung inflammation during the process of ARDS. Although the role of HMMR (CD168) in ARDS has not been studied before, we speculate that HMMR may be related with the progress of ARDS.

Despite of the results obtained above, there were some limitations in this study. Firstly, no verification experiments based on cells or tissues or joint analysis of expression profile data were performed to confirm our results, besides, the sample size was small. Secondly, due to the data themselves, there was no correction for multiple comparisons in the DEGs identification. Thirdly, the mRNA expression profile used in this study was extracted from blood leukocytes, and another important cells in ARDS, such as epithelial and endothelial cells of the lung had not been studied. Therefore, more investigations related to another cells in ARDS with experimental verification and diverse samples are needed in the further study.

## Conclusions

In conclusion, the pathways like mitotic cell cycle were closed related with the development of sepsis related ARDS. Genes including *CCNB1*, *CCNB2* and *TOP2A*, as well as transcription factors FOXM1 may be used as the novel gene therapy targets for ARDS.

## Abbreviations

ARDS: acute respiratory distress syndrome
*Ang*-*2*: *angiopoietin*-*2*
*VEGF*: *vascular endothelial growth factor*
GEO: gene expression omnibus
RMA: robust multi-array average
GO: gene ontology
MF: molecular function
BP: biological process
CC: cellular component
KEGG: Kyoto encyclopedia of genes and genomes
PPI: protein-protein interaction
NES: normalized enrichment score
*CCNB2*: *cyclin B2*
*TOP2A*: *topoisomerase II alpha*
*FOXM1*: *forkhead box protein M1*
*HMMR*: *hyaluronan*-*mediated motility receptor*
*CCNB1*: *cyclin B1*
*CCNB2*: *cyclin B2*
